# Concentration Dependent Ion-Protein Interaction Patterns Underlying Protein Oligomerization Behaviours

**DOI:** 10.1038/srep24131

**Published:** 2016-04-07

**Authors:** Helena Batoulis, Thomas H. Schmidt, Pascal Weber, Jan-Gero Schloetel, Christian Kandt, Thorsten Lang

**Affiliations:** 1 Membrane Biochemistry, Life & Medical Sciences (LIMES) Institute, University of Bonn, Bonn, Germany; 2 Life Science Informatics B-IT, Computational Structural Biology, University of Bonn, Germany

## Abstract

Salts and proteins comprise two of the basic molecular components of biological materials. Kosmotropic/chaotropic co-solvation and matching ion water affinities explain basic ionic effects on protein aggregation observed in simple solutions. However, it is unclear how these theories apply to proteins in complex biological environments and what the underlying ionic binding patterns are. Using the positive ion Ca^2+^ and the negatively charged membrane protein SNAP25, we studied ion effects on protein oligomerization in solution, in native membranes and in molecular dynamics (MD) simulations. We find that concentration-dependent ion-induced protein oligomerization is a fundamental chemico-physical principle applying not only to soluble but also to membrane-anchored proteins in their native environment. Oligomerization is driven by the interaction of Ca^2+^ ions with the carboxylate groups of aspartate and glutamate. From low up to middle concentrations, salt bridges between Ca^2+^ ions and two or more protein residues lead to increasingly larger oligomers, while at high concentrations oligomers disperse due to overcharging effects. The insights provide a conceptual framework at the interface of physics, chemistry and biology to explain binding of ions to charged protein surfaces on an atomistic scale, as occurring during protein solubilisation, aggregation and oligomerization both in simple solutions and membrane systems.

Ion-protein interactions are a key issue in fields ranging from life sciences to industrial processes. Ions are essential in controlling cell physiological processes. They play a role in oligomerization of charged rod-like biopolymers including DNA^1^, microtubules and actin^2^ and influence the aggregation of disease-associated amyloidogenic proteins^3^,^4^. In daily laboratory routines, and for the formulation of highly concentrated therapeutics^5^ they are applied to control protein solubility. Finally, self-assembling, protein-based nanomaterials are functionally modulated with the help of ions^6^.

Attempts to understand the interplay of proteins and ions date back to the 19^th^ century when Hofmeister observed that certain ions have a greater capability to precipitate (salt-out) proteins while others keep the proteins in solution (salting-in)^7^. Later, ion-selective effects concerning ion channel permeability^8^ or enzyme activity^9^ were noticed to resemble the Hofmeister series, indicating a common molecular nature underlying protein solubility and biological ion-protein interactions.

While it was formerly believed that ions (dis)order a vast solvent volume, to date it is rationalized that long-range electrostatic forces are non-existent in physiological salt solution as electrical charges are screened beyond 1 nm. Therefore, forces between ions and water are confined to the first or conceivably second hydration shell^10^. Ion-water interactions play an important role, for instance in ion-pair formation for which matching water affinities were proposed to be crucial^11^.

Transferring this concept into a cellular context requires replacing one or both ions by charged, ion-like atom groups present in biomolecules, for example charged protein residues. Different from freely diffusing ions in a salt solution, charges are confined to the biomolecules’ volume and can accumulate to a large number upon biomolecule oligomerization. Perhaps the most intriguing ion effects on biopolymers are reversible or biphasic oligomerization phenomena. When adding counterions to charged biopolymers like DNA^12^, or proteins such as lysozyme^13^ and BSA^14^, they first oligomerize or undergo phase separation. Upon further increase of the ion concentration, the behaviour is reversed and the polymers are dissolved again. The effect is supposed to result from continuous association of ions with the macromolecule. At first, ion binding neutralizes the repulsive charge component that develops upon oligomerization, allowing for higher oligomers than in the absence of counterions. Eventually, further association results in overcharging (the biomolecule-ion complex now carries the opposite net charge than the biomolecule alone) and therefore polymer (re)dispersion^14^,^15^.

Despite the ubiquitous influence of ions on protein oligomerization, the underlying molecular ion binding patterns and the effect of ions on proteins in cellular multi-component environments have not been resolved. In this study we set out to investigate whether the effects known from polymer physics also operate in complex biological systems such as the cell membrane. Moreover, by comparatively studying ion effects in wet lab experiments and in molecular dynamics simulations we aimed for unravelling ion-protein interaction patterns in molecular detail. Our experimental set-up comprises a negatively charged membrane protein (SNAP25, a member of the SNARE protein family) as a biological anion chain, and Ca^2+^ as a biologically relevant metal cation. Our data show Ca^2+^ concentration dependent biphasic oligomerization of not only soluble but also membrane-anchored SNAP25. By comparison to other ions the physicochemical requirements for ion-protein binding are addressed. The MD data reveal the interacting ionic pairs and their binding stoichiometry on an atomistic scale, and offer an explanation for the observed biphasic oligomerization behaviour which is applicable to proteins in simple solutions and in complex biological environments.

## Results and Discussion

In a first step we analysed the oligomerization behaviour of recombinant SNAP25 in response to increasing Ca^2+^ and Mg^2+^ concentrations in solution. Ca^2+^ and Mg^2+^ ions were chosen since both are highly charged and biologically relevant. For assaying oligomerization we used label-free microscale thermophoresis (MST) which takes advantage of the mass dependence of thermodiffusion. Supplementary Fig. 1 illustrates that thermodiffusion is slowed down up to intermediate calcium concentrations, suggesting SNAP25 oligomerization. However, this trend reverses again at higher concentrations, indicating a lower degree of oligomerization similar to the condition without divalent ions. The same relationship was observed for Mg^2+^, albeit less pronounced. Hence, oligomerization does not linearly, but biphasically vary with ion concentration.

In optical density measurements requiring higher SNAP25 concentrations, we observed protein precipitation (Supplementary Fig. 1), suggesting that solid phase separation can become a dominant third pathway. Notwithstanding, the data indicate that biphasic SNAP25 oligomerization is feasible, which is consistent with previous reports on other soluble proteins.

**Figure 1:**
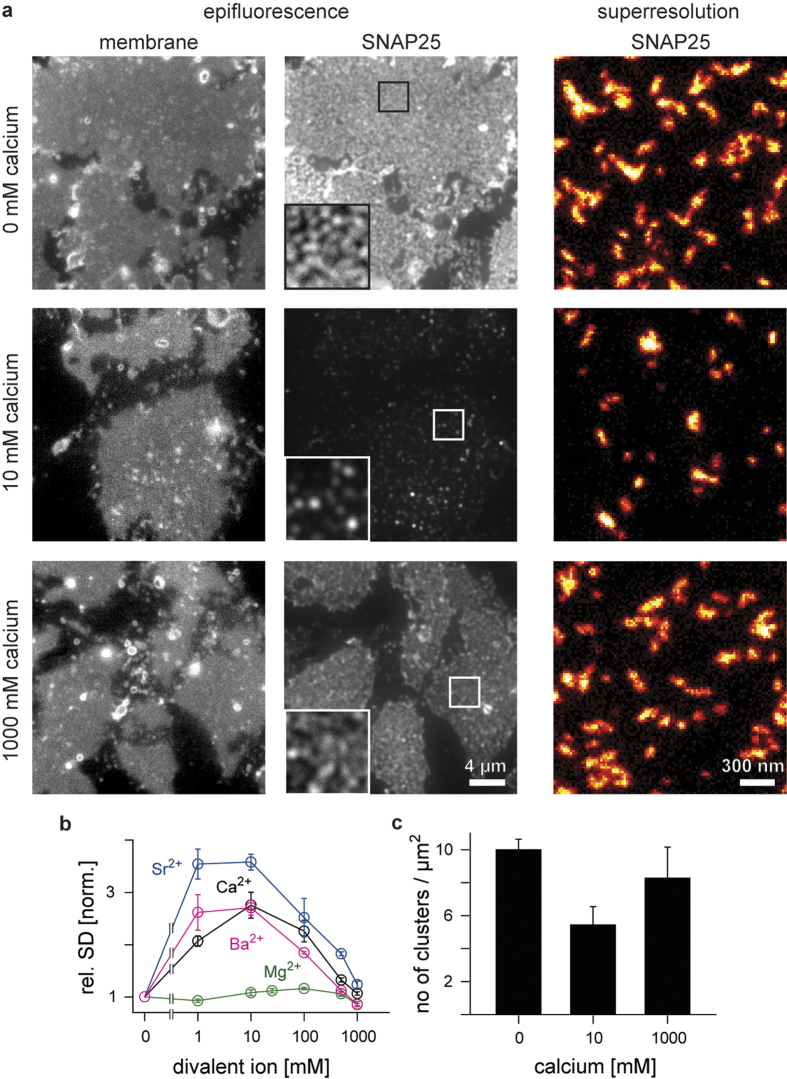
SNAP25 in native plasma membrane sheets shows biphasic clustering in response to divalent ions. PC12 plasma membrane sheets incubated at 37 °C for 10 min with the indicated concentrations of calcium, strontium, barium or magnesium chloride, fixed, immunostained for SNAP25 and analysed with epifluorescence or superresolution STED microscopy. (**a**) Membrane sheets incubated with 0, 10, or 1000 mM CaCl_2_. Epifluorescence recordings from the dye TMA-DPH (left; documenting the integrity of the membranes) and the immunostaining (middle; overviews are shown at the same, magnified insets at arbitrary scaling). Right, STED micrographs of SNAP25 immunofluorescence to which the “red hot” look up table was applied which displays increasingly brighter pixel intensities applying a colour code from black to red to yellow to white. (**b**) SNAP25 clustering was quantified by calculating the relative standard deviation (rel. SD) of the immunostaining pattern, normalized to the baseline condition which contained no divalent cations. Values are means ± s.e.m. (**c**) SNAP25 cluster density (means ± s.e.m.) resolved by superresolution STED microscopy. Cluster size was similar under all conditions (Supplementary Fig. 4).

Focussing on the more effective Ca^2+^ ion, we asked whether the protein behaves similarly in its native cell membrane environment. The cell membrane restricts movement to a two-dimensional plane thus reducing the proteins’ degrees of freedom. In addition, it is densely populated by a multitude of other protein species yielding a highly crowded environment which restricts protein mobility. SNAP25 is an integral monotopic membrane protein which is attached to the inner leaflet of the membrane via palmitoyl anchors^16^. The commonly used procedure to biochemically access intracellular proteins is the use of detergents or pore forming molecules. Those perturb the integrity of the cell membrane and may alter the proteins’ diffusional behaviour and their propensity to form aggregates. For preservation of membrane integrity, we applied a 100 ms ultrasound pulse (generating a shearing force) to the cells (neuroendocrine PC12 cells). This leaves behind large, intact basal plasma membrane areas with the inner membrane leaflet exposed to the applied buffer solution (see Fig. 1a). It has been shown that diffusion dynamics of several proteins are preserved in membrane sheets^17^,^18^, and we confirmed this for SNAP25 with fluorescence recovery after photobleaching (FRAP) experiments (Supplementary Fig. 2).

Membrane sheets were incubated in buffers with increasing concentrations of Ca^2+^. While control sheets show a bright and dispersed SNAP25 microdomain pattern, a ~75% dimmer and more punctuate staining (compare insets in Fig. 1a) emerges up to a concentration of 10 mM calcium. At higher calcium concentrations, the bright and dispersed pattern is recovered (Fig. 1a). The decrease in the average membrane sheet fluorescence intensity (Supplementary Fig. 3) is caused by epitope masking due to increased molecular crowding^19^ (for a detailed illustration of the staining intensity see Supplementary Fig. 3). We used the standard deviation of the membrane sheet fluorescence intensity as a segmentation-free analysis to quantify the heterogeneity in protein distribution. The standard deviation yields zero in case of a perfectly uniform distribution and increases upon accumulation of protein fluorescence in clusters, as both peaks and valleys in the fluorescence distribution contribute to a deviation from the mean intensity. As shown in Fig. 1b, the rel. SD reports biphasic clustering of endogenous, membrane-anchored SNAP25 which reflects the biphasic protein oligomerization observed for the isolated SNAP25 in solution. Protein clustering at intermediate and re-dispersion at high ion concentrations is not a unique feature of Ca^2+^, but is also observed for other members of the earth alkaline metals such as Sr^2+^ and Ba^2+^, and in a subtle fashion for Mg^2+^ (Fig. 1b). While the rel. SD indicates the general degree of clustering, it cannot distinguish between different underlying scenarios such as changes in cluster size/density and/or sorting of more non-clustered molecules into clusters. To complement the result obtained by the rel. SD analysis, we additionally performed a segmentation-based analysis which found that the fraction of fluorescence signal within punctuate areas peaks at 10 mM calcium (Supplementary Fig. 3).

We measured the size and the number of clusters per μm^2^ with superresolution STED microscopy. We found that upon the addition of 10 mM CaCl_2_ the cluster density is reduced by a factor of two (Fig. 1c), while the cluster size remains stable (Supplementary Fig. 4). This suggests a 2-fold tighter packing of clustered SNAP25. Considering that under this condition the fraction of clustered signal is 2.5-fold increased (Supplementary Fig. 3), this even suggests a 5-fold increase in packing density. Tighter packing is likely the result of Ca^2+^ ions attenuating the repulsive forces between negatively charged SNAP25 molecules. This explains biochemical experiments in which SNAP25 binding partner accessibility is strongly diminished by Ca^2+^ 19.

**Figure 2:**
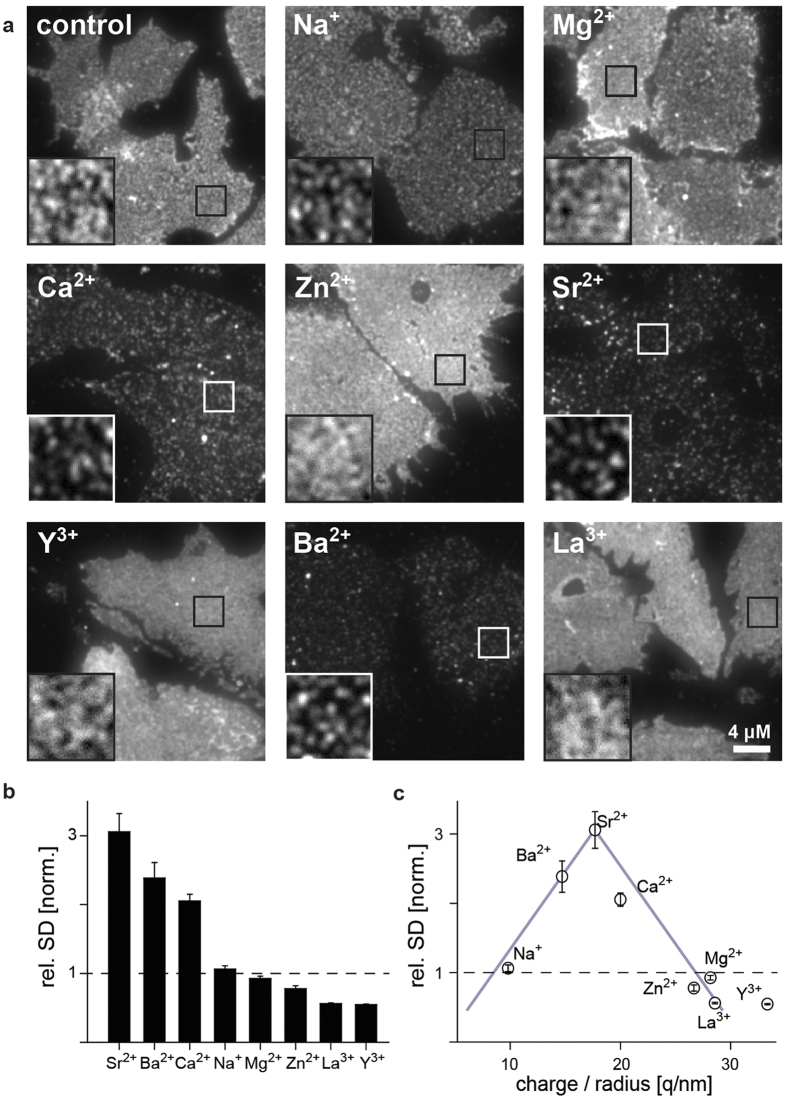
The efficacy of mono-, di- and trivalent ions on SNAP25 clustering in the cell membrane depends on ion charge-to-radius ratio. (**a**) Membrane sheets incubated at 37 °C for 10 min with 1 mM chloride salts of Na^+^, Mg^2+^, Ca^2+^, Zn^2+^, Sr^2+^, Y^3+^, Ba^2+^ and La^3+^ (sorted by atomic number), fixed, and immunostained for SNAP25. Overviews are shown at the same, magnified views at arbitrary intensity scaling. (**b**) The degree of SNAP25 clustering is expressed as rel. SD of the immunostaining pattern, normalized to the control condition. Ions are shown in decreasing order of their clustering efficacy. (**c**) Plotting values from (**b**) versus the ratio of ion charge-to-crystal radius^23^ shows a distribution resembling a volcano plot (grey lines). For relation to other ion properties see Supplementary Fig. 5. Values are means ± s.e.m.

There are numerous reports on different ions’ capacities to solubilize or aggregate proteins in solution. Here we recapitulated the effect of mono-, di- and trivalent cations on SNAP25 clustering in the native plasma membrane. The cations’ efficacy to induce SNAP25 clustering decreases from Sr^2+^ > Ba^2+^ > Ca^2+^ > Na^+^ > Mg^2+^ > Zn^2+^ > La^3+^ ~Y^3+^ (Fig. 2a,b). There is no linear relationship between efficacy and ion properties such as charge, crystal radius, volume, water viscosity coefficient, hydration energy, polarizability and softness (Supplementary Fig. 5). Instead, for the charge-to-radius ratio, hydration energy and water viscosity the graphs resemble volcano plots in which Sr^2+^ marks the maximum (Fig. 2c and Supplementary Fig. 5).

**Figure 3:**
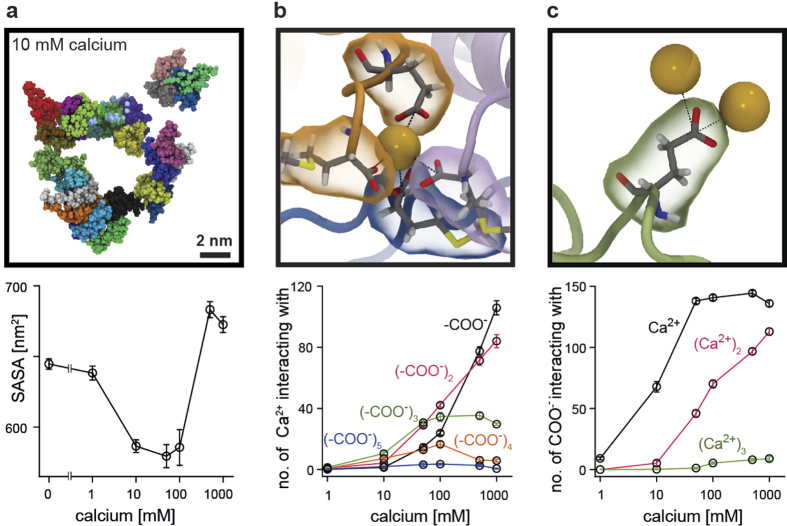
Ion-protein binding patterns depend on the calcium concentration. (**a**) Snapshot from the end of the MD simulation in the presence of 10 mM CaCl_2_ (for comparison see Supplementary Fig. 6 showing snapshots from the respective end states of all simulation runs and conditions). The solvent accessible surface area (SASA), averaged over the last 20 ns of the simulation (means ± s.e.m.) illustrating the degree of peptide clustering. (**b**) Example of an interaction between one calcium ion (yellow, van der Waals representation) and four carboxylate groups provided by three peptides (orange, magenta and blue). For clarity only protein residues providing a carboxylate group for Ca^2+^ binding are shown. The graph shows the number of interactions (averaged over the last 20 ns, means ± s.e.m.) between one calcium ion and *i* carboxylate groups (*i* = 1, 2, …, 6; indicated by different colours; for clarity *i* = 6 is not shown) as a function of Ca^2+^ concentration. (**c**) Example of an interaction between one glutamic acid carboxylate group and two calcium ions, at two different binding distances (see text). The graph shows how the number of interactions between one carboxylate group and *i* calcium ions (*i* = 1, 2, or 3) depends on the calcium concentration.

We next asked whether biphasic clustering can be retraced in an atomistic model and performed a set of 100 ns molecular dynamics (MD) simulations with 27 SNAP25 peptides, evaluating their clustering behaviour at different Ca^2+^ concentrations. In all 28 simulations (four multi-copy runs^20^,^21^ per concentration) peptides mix randomly, unbiased from their respective position in the starting configuration (Supplementary Fig. 6). Clustering was evaluated by monitoring the solvent accessible surface area (SASA) of the peptides (Fig. 3a). This readout is sensitive to initial physical contact establishment of the peptide chains as well as to structural readjustment of the proteins, which occurs during further condensation/tighter packing of oligomers (Supplementary Fig. 7). We observe similar oligomerization behaviours in the four runs performed for each calcium concentration (see Supplementary Fig. 6, and the relatively small s.e.m. of the SASA in Fig. 3a). After 100 ns the MD simulation has not yet reached equilibrium (Supplementary Fig. 7). Still, while the reaction velocities (ΔSASA/Δt) are very different within the first 10 ns, they converge to similar values at the end of the runs. Compared to wet-lab experiments, the MD system is small, and allows for the formation of one 27-mer at most (which is observed in one run at 100 mM calcium). In wet lab experiments reaction times are likely orders of magnitudes larger as much larger oligomers can form. Despite these differences, we find the same principle *in silico*, a biphasic oligomerization behaviour (Fig. 3a) remarkably similar to the wet-lab experiments. The MD data were analysed in greater detail to gain insights into the molecular basis of ion-induced clustering behaviours. Irrespective of the concentration, Ca^2+^ preferentially interacts with carboxylate groups of aspartate and glutamate side chains and of the C-terminus (Supplementary Fig. 8). Ions are located at distances of ~0.32 and ~0.38 nm from the carboxylate carbon atom. These distances resemble two coordinative binding modes between the ion and the oxygen atoms of the carboxylate group (Fig. 3c), for which Ca^2+^ has to release at least one of its hydrating water molecules. At intermediate calcium concentrations which induce clustering, the majority of bound Ca^2+^ ions interact with more than one carboxylate group (Fig. 3b). This stoichiometry implies bridging of protein carboxylates by Ca^2+^ ions is the mechanism underlying clustering. At higher Ca^2+^, an excess of ions compete for the available binding sites and saturate the individual carboxylate groups, impeding bridge formation. The remainder of Ca^2+^ ions either stay in the bulk solution or bind to already occupied carboxylates (Fig. 3c), thus entailing a local overcharging of the usually negatively charged protein residues.

In summary, the data show that both in simple aqueous systems as well as in complex membrane environments different protein oligomerization behaviours are induced depending on the Ca^2+^ concentration. In the plasma membrane, effective clustering depends on the ion parameters charge-to-radius ratio, water viscosity coefficient and free hydration energy (per coordinated water molecule). The close proximity between Ca^2+^ and carboxylate observed in the MD supports the notion that ion-bridge formation involves release of a water molecule, or more precisely, the exchange of oxygen from water for oxygen from a carboxylate group, and thus corroborates the importance of the hydration energy for clustering. Ligand exchange is only energetically favorable (ΔH < 0) if the metal hydration enthalpy is lower than the ion-carboxylate binding enthalpy. The Mg^2+^ ion has a comparably high hydration enthalpy and a 3,000-fold lower water exchange rate than a Ca^2+^ ion^22^. This hampers binding of Mg^2+^ to carboxylate groups and all therefrom resulting effects, which explains why Mg^2+^ is less potent than Ca^2+^ at inducing clustering. Insuperable free energies of ionic hydration can likewise explain why we observe no clustering effects for Zn^2+^, La^3+^ and Y^3+^ (−ΔG_hydr_/CN > 300 kJ/mol; Supplementary Fig. 5). In addition to a suitable hydration energy, a certain charge density is required to overcome repulsions between the negatively charged proteins during clustering, explaining why Na^+^ shows no effect. The data are also in accordance with Collin’s “law of matching water affinities”^11^. According to this theory a similarity in water affinity, which is reflected by the viscosity coefficient, predicts preferential ion pair formation. In line with this prediction, the water viscosity coefficient of acetate anions (0.246 cm^3^/mol)^23^ is comparable to that of the ions which are effective at inducing SNAP25 clustering (Supplementary Fig. 5).

In conclusion, the MD simulations show how the stoichiometry of the calcium-carboxylate interactions depends on ion concentration and determines protein oligomerization behaviour. At low concentrations, ion bridges between calcium ions and negatively charged protein carboxylate groups link individual proteins to each other, and counterion binding also compensates for charge accumulation occurring during oligomerization. However, excessive counterion binding at high concentrations implies saturation and overcharging of the carboxylate groups, impedes bridge formation and evokes protein repulsion and (re-)dispersion.

To probe the experimental systems’ constraints and to recognize parallels in their behaviours, we extended the calcium concentration into a range far higher than found in body fluids or the intracellular milieu. Therefore it can be argued that the insights are of theoretical interest, relevant for industrial processes, drug delivery, bionanomaterial science, or *ex vivo* conditions. Still, while dispersal of oligomers by overcharging is unlikely *in vivo*, Ca^2+^ salt bridges will readily form in the extracellular milieu that contains ~1.5 mM free Ca^2+^ 24. In the MD simulation at 1 mM Ca^2+^, all three available ions (Supplementary Table 1) are involved in salt bridge formation in 94% of the analysed trajectory frames. As only few salt bridges are sufficient for oligomerization, negatively charged proteins in the blood, extracellular fluid or on the extracellular surface will experience oligomerization based on Ca^2+^ salt bridges. In few cases, even intracellular processes may be controlled by salt bridges. During synaptic transmission, calcium channel opening can locally generate Ca^2+^ levels in synaptic terminals of 100 – 200 μM^25^, and indeed depolarization-induced calcium influx causes SNAP25 crowding^19^.

In conclusion, we explored phenomena known from polymer physics and solution chemistry in the native cell membrane. Intriguingly, while there are several differences between a simple aqueous environment and a crowded two-dimensional membrane, general principles like ion-induced protein clustering and protein overcharging appear to hold true from polymer science to membrane biology. Based on the revealed atomistic binding patterns, the study provides a conceptual framework for the understanding of different ion-induced protein oligomerization behaviours in simple and complex molecular environments with implications for industrial and biological processes.

## Methods

### Cloning, expression and purification of SNAP25

SNAP25 is 206 amino acids in length and belongs to the SNARE protein family with a biological function in synaptic vesicle exocytosis^16^. It is attached to the intracellular membrane leaflet via palmitoyl anchors. At pH 7.4, the side chains of aspartic and glutamic acid are negatively charged, whereas arginine, lysine and histidine carry positive charges (histidine less so, as its side chain has a pK_a_ of ~6). In this respect, apart from the oppositely charged N- and C-termini, SNAP25 contains 43 negatively and 30 positively charged amino acid residues, and therefore was used as a model for an overall negatively charged biopolymer. The sequence of rat SNAP25B was amplified from a previously used construct (GFP-labelled SNAP25 26) by polymerase chain reaction (PCR) using primers with restriction sites for *BamHI* (forward primer) and *EcoRI* (reverse primer). The SNAP25 sequence was subcloned into the pGEM-T easy vector system (catalogue no. A1360, Promega) by TA cloning, and subsequently subcloned into the expression vector pGEX-6P1 (GE Healthcare Life Sciences) using the *BamHI* and *EcoRI* restriction sites. The construct was verified by sequencing using the SNAP25B sequence (AB003992) as a reference. The construct was transformed into Rosetta(DE3)pLysS (Merck) and after induction with 1 mM IPTG, soluble SNAP25-GST was expressed overnight at 18 °C, yielding the non-palmitoylated form of the protein. For purification, bacterial pellets were sonicated in binding buffer (50 mM Tris-HCl, 150 mM NaCl, 1 mM EDTA, 1x cOmplete (Roche, Basel, Switzerland), 1 mM PMSF, 1 mM DTT, pH 7.4) containing 100 μg/ml lysozyme (Carl Roth) and 2 u/ml DNAse I (NEB, Ipswich, MA). After a centrifugation step, cleared lysates were bound to equilibrated Glutathione Sepharose 4B beads (GE Healthcare) rolling at 4 °C overnight. After several washing steps with cleavage buffer (50 mM TrisHCl, 150 mM NaCl, 1 mM EDTA, 1 mM DTT, pH 7.4), the GST-tag was cut off on the column by incubation with 380 units PreScission Protease (GE Healthcare) for 5 hours at 4 °C in the same buffer. SNAP25 was recovered from the column in cleavage buffer. The sample was concentrated and buffer was exchanged to TBS (50 mM Tris-HCl, 150 mM NaCl, pH 7.4) containing 1 mM DTT and 10% [v/v] glycerol using Amicon Ultra-15 centrifuge filters (10 kDa cut-off, Merck Millipore). The aliquoted samples were frozen in liquid nitrogen and stored at −80 °C.

### Microscale thermophoresis (MST)

Titration series with 10 μM SNAP25 and CaCl_2_ or MgCl_2_ (Carl Roth) concentrations varying between 0 and 1000 mM were prepared in TBS containing 0.1% [v/v] Pluronic F-127 (Sigma, St. Louis, MO). Approximately 3 μl were loaded into NT.LabelFree Standard treated capillaries (Nanotemper). MST experiments were performed at 40% MST (infra-red laser) power and 30% LED power at 37 °C using the Monolith NT.LabelFree Instrument (Nanotemper). Ratios between normalized initial fluorescence and after temperature-jump and thermophoresis were calculated and averaged from five to nine independent runs (half of the runs were incubated for 30 minutes at room temperature (RT) before the measurement while the other half were directly measured). In few cases MST traces showed signs of protein precipitation, or large deviations from the average initial fluorescence, and were not included in the analyses.

### Generation, treatment and staining of PC12 membrane sheets

Membrane sheets were generated by application of a brief ultrasound pulse to glass-adhered PC12 cells (see below), removing the apical membrane and the cytosol and leaving behind the basal plasma membrane attached to a coverslip. They provide quick access to the inner leaflet of the plasma membrane and allow for higher signal-to-noise ratios in fluorescence microscopy imaging.

PC12 cells (a kind gift of Rolf Heumann, University of Bochum, Germany) were maintained at 37 °C with 5% CO_2_, and cultured in DMEM with 4.5 g/l glucose and L-glutamine (PAN biotech), supplemented with 10% [v/v] horse serum, 5% [v/v] fetal calf serum (both Biochrom AG), and 1% [v/v] penicillin/streptomycine (PAN biotech). Cells were plated onto poly-L-lysine-coated glass coverslips (6*10^5 ^cells per coverslip in a six-well plate) and used the following day for experiments. For generation of membrane sheets, cells were placed in ice-cold Hepes/KCl/EGTA buffer (140 mM KCl, 10 mM EGTA, 20 mM Hepes-KOH, pH 7.2), and a 100 ms ultrasound pulse was applied. The sheets were washed once with ice-cold Hepes/KCl buffer (140 mM KCl, 20 mM Hepes-KOH, pH 7.2), and then incubated for 10 minutes at 37 °C with either Hepes/KCl/EGTA buffer (EGTA was added for chelating traces of divalent and trivalent ions), or CaCl_2_, SrCl_2_, BaCl_2_, MgCl_2_, NaCl (all from Carl Roth), ZnCl_2_, YCl_3_, or LaCl_3_ (all from Sigma) at the indicated salt concentrations in Hepes/KCl buffer. Then membrane sheets were fixed in 4% [w/v] paraformaldehyde in Hepes/KCl for 45 minutes at RT. The PFA was quenched with 50 mM NH_4_Cl in PBS (137 mM NaCl, 2.7 mM KCl, 10 mM Na_2_HPO_4_, 1.76 mM KH_2_PO_4_, pH 7.4) for 15 min, followed by several washes in PBS. The membrane sheets were then blocked for 1 h with 3% [w/v] BSA in PBS (BSA-PBS), and incubated with the primary antibody mouse anti-SNAP25 (clone 71.1, Synaptic Systems, diluted 1:200 in BSA-PBS overnight at 4 °C. After three PBS washing steps, sheets were incubated with the secondary antibody Alexa Fluor 594-labelled donkey anti-mouse (ThermoFisher, catalog no. A-21203, diluted 1:200 in BSA-PBS) for 1 h at RT. After renewed washing, the membrane was counterstained using TMA-DPH ((1-(4-tri-methyl-ammonium-phenyl)-6-phenyl-1,3,5-hexatriene-p-toluenesulfonate) ThermoFisher) for imaging the membrane sheets in the blue channel in epifluorescence microscopy. For STED microscopy samples, the same protocol was applied except that the first antibody was diluted 1:100 and as secondary antibody Star 635p-labelled goat anti-mouse (Abberior, catalog no. 2-0002-007-5, diluted 1:500 in BSA-PBS) was used. The Star 635p fluorophore was used since it provides high resolution when using a 775 nm laser for STED^27^ and is sufficiently photostable at the high laser intensities applied for STED microscopy. As membrane counterstain 0.5 μg/ml fastDiO in PBS (Thermo Fisher Scientific) was applied for 10 min at RT followed by washing in PBS and embedding in ProLong Gold Antifade Mountant (Thermo Fisher Scientific). Prior to imaging, samples were stored at RT. Membrane counterstains were used to identify damaged membranes during imaging and analysis. Different membrane stains, TMA-DPH and fastDiO, were chosen due to different spectral requirements of the epifluorescence and STED microscope equipment.

### Microscopy and image analysis

For epifluorescence, membrane sheets were imaged immediately after staining in a microscopy chamber in PBS at RT using the Zeiss Axio Observer D1 epifluorescence microscopy (Zeiss) equipped with a Zeiss Plan-Apochromat 100x/NA 1.4 oil immersion objective. Membrane sheets were selected based on intact TMA-DPH appearance. Images were acquired with a cooled digital 12 bit CCD camera (Seniscam QE; PCO AG) with 1376 × 1040 pixels and a pixel size of 6.45 × 6.45 μm using the CamWare (V 3.01) software (PCO AG). The filter sets F36-503 and the F11-000 (both AHF Analysetechnik, Tübingen, Germany) were used for acquiring images of the TMA-DPH and the Alexa 594 fluorescence, respectively. For STED microscopy a 4-channel easy3D superresolution STED microscope (Abberior Instruments) equipped with an Olympus UPlanSApo 100x/NA 1.4 objective was used. Membrane sheets were selected based on intact fastDiO appearance. The fastDiO membrane counterstain was excited with a pulsed 488 nm laser and detected with a 500–520 nm filter. For imaging of SNAP25 immunofluorescence, Star 635p was excited with a pulsed 561 nm laser, depleted with a pulsed 775 nm STED laser with 1.25 ns gating and detected with a 650–720 nm filter. The channels were scanned with a pixel size of 20 nm × 20 nm and 20 μs dwell time using the software Imspector (Abbrerior Instruments).

Images were analysed with the ImageJ software. A region of interest (50 pixel × 50 pixel for epifluorescence and 150 pixel × 150 pixel for STED microscopy) was placed in the membrane reference image (recorded in the TMA-DPH or fastDiO channel) and then transferred to the SNAP25 immunostaining recording (Alexa 594 or Star 635p channel). For epifluorescence microscopy also background values were taken by placing the ROI next to the membrane sheets. For epifluorescence, the background corrected mean fluorescence intensity and its relative standard deviation were determined. For STED microscopy, the image J “find maxima” tool was used for counting clusters applying the same noise level for all recordings. To estimate cluster radius, the ROI was shifted pixel-wise to the right, and the Pearson correlation coefficient (PCC) between the shifted image and the original image was plotted against the shift distance. The autocorrelation curves of all ROIs from a certain condition and day were averaged. The curves were fitted with a polynomial function, and the 50% decay value, which reflects the average object radius in the image, was calculated in Origin (v8).

All values are given as means ± s.e.m. (epifluorescence, n = 3–7 independent experiments, each comprising 16–133 membrane sheets per condition, Fig. 2b contains also the 1 mM values from Fig. 1b; STED microscopy, n = 4 independent experiments, 10–16 membrane sheets per condition).

### Molecular dynamics simulations and analysis

To maintain realistic computation times, a peptide of 30 amino acid length was used instead of full length SNAP25. The peptide (Leu 35–Met 64) was extracted from the crystal structure of the assembled SNARE complex (PDB-ID: 1SFC, chain C), in which it forms the layers −3 to +5 of the *N*-terminal SNAP25B SNARE motif (SN1)^28^. This peptide conveys a net charge of −6. After a steepest descent energy minimization *in vacuo*, 27 copies of these helical peptides were placed in random orientations and solvated in a 160 mM NaCl/TIP3P water^29^ environment. Additional counterions were added to neutralize the overall system charge. Seven MD systems were set up containing CaCl_2_ concentrations of 0, 1, 10, 50, 100, 500, and 1000 mM, respectively (see Supplementary Table 1 for detailed system data). After a second steepest descent energy minimization, each system was simulated in n = 4 independent 100 ns MD runs (*multi-copy* approach^20^,^21^). The AMBER99SB-ILDN *all-atom* force field^30^ was used. The Berendsen weak coupling was used to maintain a temperature of 310 K (*τ* = 1.6 ps) for the protein and the solvent, separately^30^. The analogue barostat^31^ was used to adjust the pressure to a reference value of 1.013 bar every 4 ps using isotropic coupling. Long range interactions were handled using the smooth Particle Mesh Ewald methodology^32^, while a *twin-range cut-off* of 1.0 nm was used to handle van der Waals interactions. All bonds between covalently linked atoms were held constant using the LINCS algorithm^33^, allowing a MD integration step width of 2 fs.

MD simulations were performed using GROMACS 5.0.1^34^,^35^, on a cluster node of a CPU/GPU computer system (2x Intel Xeon E5-2670 CPUs (8 cores, 2.6 GHz), 64 GByte RAM and one nVIDIA K20X GPU (2,496 core, 5 GByte RAM)) using one 8-core CPU (16 threads), 4 GByte RAM).

For monitoring the oligomerization properties, the overall protein solvent accessible surface area (SASA) was analysed for each frame of the individual trajectories using the GROMACS tool *gmx sasa* (0.14 nm solvent probe radius)^34^,^36^. The SASA average was computed referring to the last 20 ns of the four multi-copy runs of each system. Values are given as means ± s.e.m. (n = four simulations for each condition).

To identify the preferred interaction partners of Ca^2+^ ions, the radial distribution functions (RDFs) between Ca^2+^ and the carbon atoms of the carboxylate groups of Asp, Glu, and the C-terminus, oxygen atoms of the protein backbone and of the side chain carbonyl group of Gln, oxygen atoms of the hydroxyl groups of Ser and Thr, and nitrogen atoms of the side chain amino groups of Gln, Arg, and Lys were determined using the program *gmx rdf* (see Supplementary Fig. 8a). The RDFs yielded increased probabilities for locations ranging from 0.25 (for carbonyl oxygens) to 0.5 nm (for amino nitrogens). On the basis of these distance parameters we set individual thresholds for direct interactions between Ca^2+^ and the respective atoms (see Supplementary Fig. 8). The interaction patterns between Ca^2+^ ions and the carboxylate groups were analyzed by counting direct interactions (within a 0.4 nm threshold) for the individual trajectory frames of the last 20 ns. This analysis was based on the GROMACS program *gmx mindist*. The data were then grouped according to the type of interaction (one Ca^2+^ interacting with (COO^−^)_*i*_, *i* = 1, 2, 3, …, 10) and normalized to the number of analysed trajectory frames. An analogous procedure was used to monitor the occurrence frequencies of one COO^−^ group with *i* Ca^2+^ ions. Values are given as means ± s.e.m. (n = four simulations for each condition).

## Additional Information

**How to cite this article**: Batoulis, H. *et al*. Concentration Dependent Ion-Protein Interaction Patterns Underlying Protein Oligomerization Behaviours. *Sci. Rep*. **6**, 24131; doi: 10.1038/srep24131 (2016).

## Supplementary Material

Supplementary Information

## References

[b1] Ma C. & Bloomfield V. A. Condensation of supercoiled DNA induced by MnCl2. Biophys. J. 67, 1678–1681 (1994). 7819499 10.1016/S0006-3495(94)80641-1 PMC1225529

[b2] Tang J. X., Wong S., Tran P. T. & Janmey P. A. Counterion induced bundle formation of rodlike polyelectrolytes. Berichte Bunsenges. Für Phys. Chem. 100, 796–806 (1996).

[b3] Alies B., Hureau C. & Faller P. The role of metal ions in amyloid formation: general principles from model peptides. Metallomics 5, 183 (2013). 23416948 10.1039/c3mt20219d

[b4] Breydo L. & Uversky V. N. Role of metal ions in aggregation of intrinsically disordered proteins in neurodegenerative diseases. Metallomics 3, 1163–1180 (2011). 21869995 10.1039/c1mt00106j

[b5] Salinas B. A. . Understanding and modulating opalescence and viscosity in a monoclonal antibody formulation. J. Pharm. Sci. 99, 82–93 (2010). 19475558 10.1002/jps.21797 PMC3927449

[b6] Dannhauser P. N., Platen M., Böning H. & Schaap I. a. T. Durable protein lattices of clathrin that can be functionalized with nanoparticles and active biomolecules. Nat. Nanotechnol. 10, 954–957 (2015). 26367107 10.1038/nnano.2015.206

[b7] Hofmeister F. Zur Lehre von der Wirkung der Salze. Arch. Für Exp. Pathol. Pharmakol. 24, 247–260 (1888).

[b8] Madhavan N., Robert E. C. & Gin M. S. A Highly Active Anion-Selective Aminocyclodextrin Ion Channel. Angew. Chem. Int. Ed. 44, 7584–7587 (2005). 10.1002/anie.200501625 16247816

[b9] Zhao H. Effect of ions and other compatible solutes on enzyme activity, and its implication for biocatalysis using ionic liquids. J. Mol. Catal. B Enzym. 37, 16–25 (2005).

[b10] Omta A. W., Kropman M. F., Woutersen S. & Bakker H. J. Negligible Effect of Ions on the Hydrogen-Bond Structure in Liquid Water. Science 301, 347–349 (2003). 12869755 10.1126/science.1084801

[b11] Collins K. D. Charge density-dependent strength of hydration and biological structure. Biophys. J. 72, 65–76 (1997). 8994593 10.1016/S0006-3495(97)78647-8 PMC1184297

[b12] Pelta J., Livolant F. & Sikorav J.-L. DNA Aggregation Induced by Polyamines and Cobalthexamine. J. Biol. Chem. 271, 5656–5662 (1996). 8621429 10.1074/jbc.271.10.5656

[b13] Zhang Y. & Cremer P. S. The inverse and direct Hofmeister series for lysozyme. Proc. Natl. Acad. Sci. 106, 15249–15253 (2009). 19706429 10.1073/pnas.0907616106 PMC2741236

[b14] Zhang F. . Reentrant Condensation of Proteins in Solution Induced by Multivalent Counterions. Phys. Rev. Lett. 101, 148101 (2008). 18851577 10.1103/PhysRevLett.101.148101

[b15] Nguyen T. T. & Shklovskii B. I. Complexation of DNA with positive spheres: Phase diagram of charge inversion and reentrant condensation. J. Chem. Phys. 115, 7298–7308 (2001).

[b16] Rizo J. & Südhof T. C. The Membrane Fusion Enigma: SNAREs, Sec1/Munc18 Proteins, and Their Accomplices—Guilty as Charged? Annu. Rev. Cell Dev. Biol. 28, 279–308 (2012). 23057743 10.1146/annurev-cellbio-101011-155818

[b17] Frick M., Schmidt K. & Nichols B. J. Modulation of lateral diffusion in the plasma membrane by protein density. Curr. Biol. CB 17, 462–467 (2007). 17331726 10.1016/j.cub.2007.01.069

[b18] Sieber J. J. . Anatomy and Dynamics of a Supramolecular Membrane Protein Cluster. Science 317, 1072–1076 (2007). 17717182 10.1126/science.1141727

[b19] Zilly F. E. . Ca^2+^ induces clustering of membrane proteins in the plasma membrane via electrostatic interactions. EMBO J. 30, 1209–1220 (2011). 21364530 10.1038/emboj.2011.53 PMC3094119

[b20] Caves L. S., Evanseck J. D. & Karplus M. Locally accessible conformations of proteins: multiple molecular dynamics simulations of crambin. Protein Sci. Publ. Protein Soc. 7, 649–666 (1998). 10.1002/pro.5560070314 PMC2143962 9541397

[b21] Koch D. C., Schmidt T. H., Sahl H.-G., Kubitscheck U. & Kandt C. Structural dynamics of the cell wall precursor lipid II in the presence and absence of the lantibiotic nisin. Biochim. Biophys. Acta BBA - Biomembr. 1838, 3061–3068 (2014). 10.1016/j.bbamem.2014.07.024 25128154

[b22] Maguire M. E. & Cowan J. A. Magnesium chemistry and biochemistry. Biometals 15, 203–210 (2002). 12206387 10.1023/a:1016058229972

[b23] Marcus Y. Ion properties. (Marcel Dekker, 1997).

[b24] Carafoli E. Intracellular Calcium Homeostasis. Annu. Rev. Biochem. 56, 395–433 (1987). 3304139 10.1146/annurev.bi.56.070187.002143

[b25] Rizzuto R. & Pozzan T. Microdomains of Intracellular Ca^2+^: Molecular Determinants and Functional Consequences. Physiol. Rev. 86, 369–408 (2006). 16371601 10.1152/physrev.00004.2005

[b26] Halemani N. D., Bethani I., Rizzoli S. O. & Lang T. Structure and Dynamics of a Two-Helix SNARE Complex in Live Cells. Traffic 11, 394–404 (2010). 20002656 10.1111/j.1600-0854.2009.01020.x

[b27] Wurm C. A. . Novel red fluorophores with superior performance in STED microscopy. Opt. Nanoscopy 1, 7 (2012).

[b28] Sutton R. B., Fasshauer D., Jahn R. & Brunger A. T. Crystal structure of a SNARE complex involved in synaptic exocytosis at 2.4 Å resolution. Nature 395, 347–353 (1998). 9759724 10.1038/26412

[b29] Jorgensen W. L., Chandrasekhar J., Madura J. D., Impey R. W. & Klein M. L. Comparison of simple potential functions for simulating liquid water. J. Chem. Phys. 79, 926–935 (1983).

[b30] Lindorff-Larsen K. . Improved side-chain torsion potentials for the Amber ff99SB protein force field. Proteins Struct. Funct. Bioinforma. 78, 1950–1958 (2010). 10.1002/prot.22711 PMC2970904 20408171

[b31] Berendsen H. J. C., Postma J. P. M., van Gunsteren W. F., DiNola A. & Haak J. R. Molecular dynamics with coupling to an external bath. J. Chem. Phys. 81, 3684–3690 (1984).

[b32] Essmann U. . A smooth particle mesh Ewald method. J. Chem. Phys. 103, 8577–8593 (1995).

[b33] Hess B. P-LINCS: A Parallel Linear Constraint Solver for Molecular Simulation. J Chem Theory Comput 4, 116–122 (2007). 10.1021/ct700200b 26619985

[b34] Van Der Spoel D. . GROMACS: Fast, flexible, and free. J. Comput. Chem. 26, 1701–1718 (2005). 16211538 10.1002/jcc.20291

[b35] Hess B., Kutzner C., van der Spoel D. & Lindahl E. GROMACS 4: Algorithms for Highly Efficient, Load-Balanced, and Scalable Molecular Simulation. J. Chem. Theory Comput. 4, 435–447 (2008). 26620784 10.1021/ct700301q

[b36] Eisenhaber F., Lijnzaad P., Argos P., Sander C. & Scharf M. The double cubic lattice method: Efficient approaches to numerical integration of surface area and volume and to dot surface contouring of molecular assemblies. J. Comput. Chem. 16, 273–284 (1995).

