# Supplementary material for: Concentration Dependent Ion-Protein Interaction Patterns Underlying Protein Oligomerization Behaviours
Source: Sci Rep. 2016 Apr 7;6:24131. doi: 10.1038/srep24131 (PMC4823792; doi:10.1038/srep24131)
Supplement: Supplementary Information [file srep24131-s1.pdf]

Supplementary Information

Concentration Dependent Ion-Protein Interaction Patterns Underlying Protein Oligomerization Behaviours

Helena Batoulis[1], Thomas H. Schmidt[1], Pascal Weber[1], Jan-Gero Schloetel[1], Christian Kandt[2] and Thorsten Lang[1]

[1] Membrane Biochemistry, Life & Medical Sciences (LIMES) Institute, University of Bonn, Bonn, Germany

[2] Life Science Informatics B-IT, Computational Structural Biology, University of Bonn, Germany. Current address: Bonn-Rhein-Sieg University of Applied Sciences, Dept. of Electrical/Mechanical Engineering and Tech. Journalism, Grantham Allee 20, 53757 Sankt Augustin, Germany

Correspondence and requests for materials should be addressed to T.L. (email: thorsten.lang@uni-bonn.de)

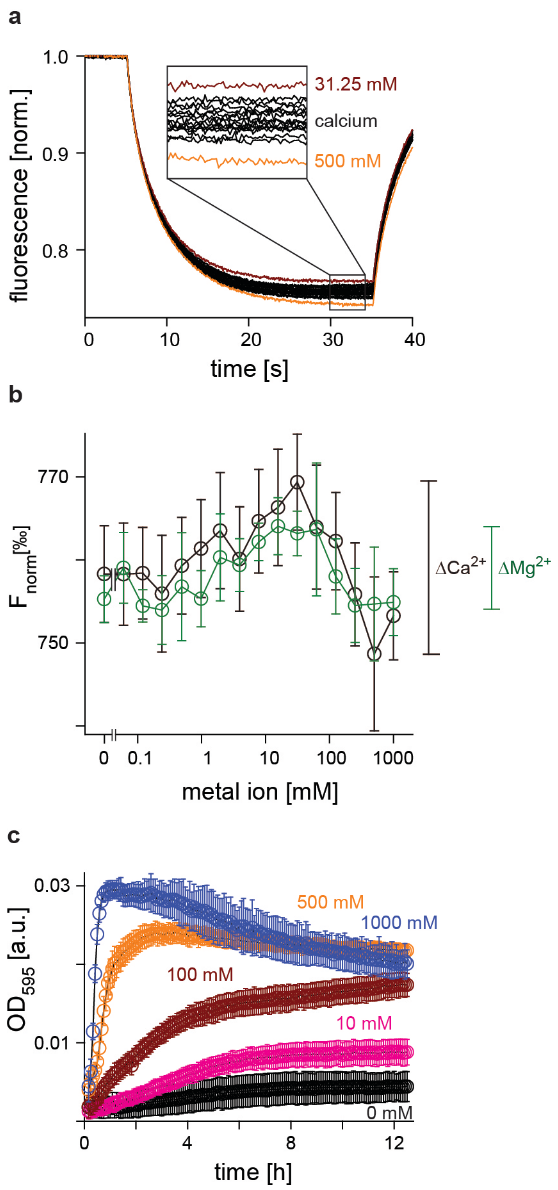

Supplementary Figure 1. Ion concentration dependent oligomerization behaviour of SNAP25 in solution.

(**a** and **b**) Oligomerization of SNAP25 measured by label-free MST with serial dilutions of $CaCl_2$ or $MgCl_2$ varied from 61 µM to 1000 mM, at a fixed SNAP25 concentration of 10 µM at 37°C. (**a**) Normalized SNAP25 tryptophan fluorescence traces, illustrating the temperature-jump and the thermophoresis of SNAP25 at different calcium concentrations in one experiment. Traces yielding the minimum (500 mM $CaCl_2$) and maximum (31.25 mM $CaCl_2$) values are colored in purple and orange, respectively. (**b**) Normalized tryptophan fluorescence after thermophoresis and temperature jump (F_{norm}, expressed in per mille), obtained from experiments as illustrated in (**a**), averaged and plotted versus the concentration of the divalent ion (calcium, black; magnesium, green). Values are mean ± s.d. (n = 5 - 9). The dynamic ranges of signal change for Ca^{2+} and Mg^{2+} are indicated by brackets. An increase in F_{norm}

indicates slower thermodiffusion. The transition from left to right suggests the presence of low oligomers in the absence of divalent ions (reflecting the known self-association of SNAREs), forming higher oligomers at middle concentrations that disperse to possibly monomeric SNAP25 at maximal Ca^{2+}. Note that the solutions' viscosity increases with ion concentration. This potentially causes underestimation of the drop in F_{norm} observed at highest ion concentrations. (**c**) Effect of calcium on SNAP25 assayed by optical density measurements. Example showing 70 µM SNAP25 incubated with the indicated Ca^{2+} concentrations in TBS at 37 °C. The optical density of the solution was determined with a Tecan plate reader (384 multi-well plate, loaded with 50 µl solutions) at 595 nm. Values are corrected by blank values from buffers containing the respective Ca^{2+} concentrations. In case of high Ca^{2+}, the initial precipitation phase is apparently reversible. Values are mean + s.d. (n = 4 – 5 technical replicates from one dilution series). Similar experiments with slightly different set-ups were performed, yielding essentially the same observation.

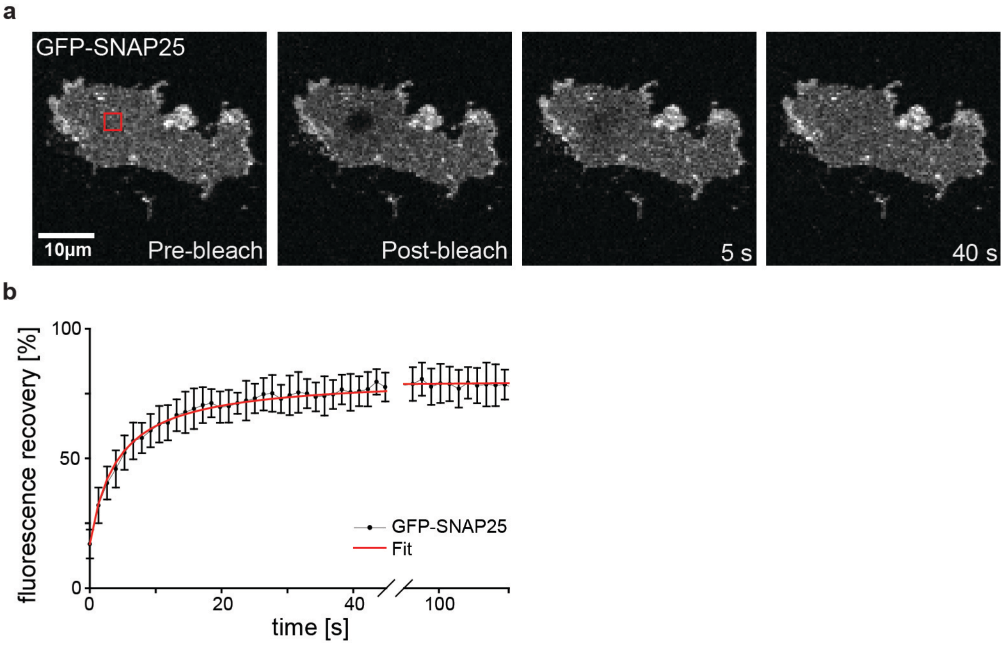

Supplementary Figure 2. SNAP25 diffusion in native cell membrane sheets.

(**a**) Confocal micrographs taken from a FRAP (fluorescence recovery after photobleaching) sequence of a membrane sheet from a PC12 cell expressing GFP-labelled SNAP25. (**b**) Average recovery trace from one representative experiment. Error bars show the standard deviation of the mean (n = 12 membrane sheets). From the fit of the averaged recovery trace (red) the half time of recovery ($t_{1/2}$) was determined, for four independent experiments (8 – 12 membrane sheets per experiment). The diffusion coefficient D was calculated from the individual half times taking into account the ROI size and a correction factor for the ROI geometry as previously described[1]. This diffusion coefficient ($D = 0.19 \pm 0.02$ µm^2/s; n = 4) is similar to a previously published value ($D = 0.24$ µm^2/s) measured in living cells[2].

For this experiment, a monomeric variant of mEGFP fused N-terminally to the sequence of full length rat SNAP-25B[3] was transfected using the Neon® Transfection System (Thermo Fisher Scientific). After 48 h membrane sheets were generated in ice-cold buffer (20 mM HEPES-KOH, 120 mM KGlu, 20 mM KAc, 10 mM EGTA, pH, 7.2) in which they remained for direct analysis by a confocal laser scanning microscope at 37°C, performed essentially as previously described[1]. For maximum bleaching efficiency the 488 nm laser in combination with a 405 nm laser were set to their maximum intensity. Pixel size was adjusted to 414 nm and the ROI size for bleaching was 7 pixels x 7 pixels (2.9 µm x 2.9 µm). Fluorescence recovery was monitored over a time period of about 2 min and recovery traces were determined and analysed as previously described[1].

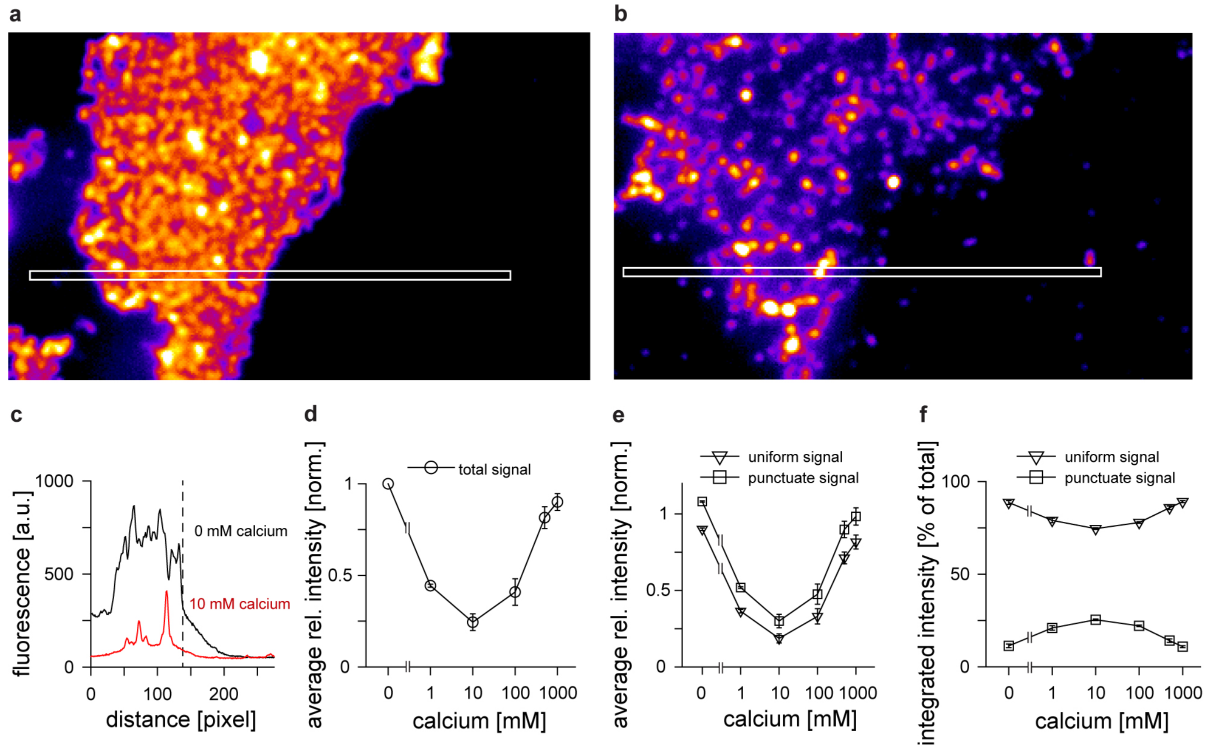

Supplementary Figure 3. SNAP25 immunostaining intensity varies biphasically with the calcium concentration.

(**a**, **b** and **c**) Illustration of the immunofluorescence intensity signal distribution by employing a "fire" lookup table at arbitrary scaling (which displays increasingly brighter pixels from blue, to red to yellow to white) in an image from the control condition (**a**) and at 10 mM calcium (**b**). (**c**) Fluorescence intensity values measured in the linescans illustrated in **a** and **b** (white boxes). The dotted line marks the edges of the membrane sheets. (**d)** Average immunofluorescence intensity (mean ± s.e.m.) of the total SNAP25 signal plotted against the calcium concentration and normalized to the control condition (without calcium). The decrease in fluorescence intensity at intermediate calcium concentrations indicates epitope masking due to a higher SNAP25 packing density and/or a conformational change, as previously described[1]. (**e** and **f**) The staining pattern was segmented into uniform and punctuate signal employing the following algorithm. First, a Gaussian blur filter was applied, and a variable local threshold (central pixel intensity > mean intensity of a 5 x 5 pixel area around the central pixel plus four percent of the mean intensity within the total membrane sheet ROI) was computed to define punctuate areas. These areas were enlarged using a single dilation to ensure that flanks of punctuate signals are included. Subsequently these areas were transferred to the original, non-processed image for determining the average fluorescence intensity within the punctuate areas (squares in **e**) and within uniform areas next to them

(triangles in **e**) (mean ± s.e.m., normalized to the control in **d)**. (**f**) Amount of fluorescence (mean ± s.e.m.) originating from uniformly distributed (triangles in **f**) and clustered (squares in **f**) signal expressed as a percentage of the total integrated membrane fluorescence (integrated uniform signal = total area * average uniform signal intensity; integrated punctuate signal = punctuate area * (average punctuate intensity - average uniform intensity)). The calcium induced changes in clustering are similar to the changes in the rel. S.D. (Fig. 1b), suggesting that the two different types of analysis are in good agreement. The fraction of the clustered signal cannot directly be translated into the fraction of clustered molecules, since it is a strong underestimate for several reasons. First, some clusters are not resolved. Second, each cluster produces a spot with a Gaussian intensity distribution. At high cluster densities the bases of the Gaussians partially overlap increasing the apparent uniform signal (see also **c**). Third, antibodies may preferentially stain the uniform SNAP25 pool as steric inaccessibility is less likely to occur.

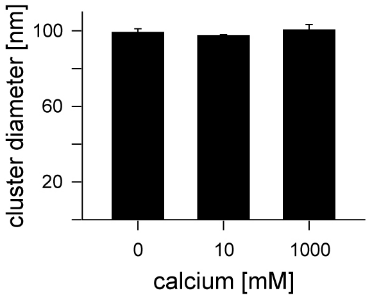

Supplementary Figure 4. SNAP25 cluster diameter determined by STED microscopy. Cluster sizes (mean ± s.e.m.) measured in SNAP25 immunostainings imaged with STED microscopy. Representative STED images are shown in Fig. 1a (right column). In STED microscopy we did not observe a uniformly distributed signal pool, possibly due to the lower sensitivity of this imaging technique.

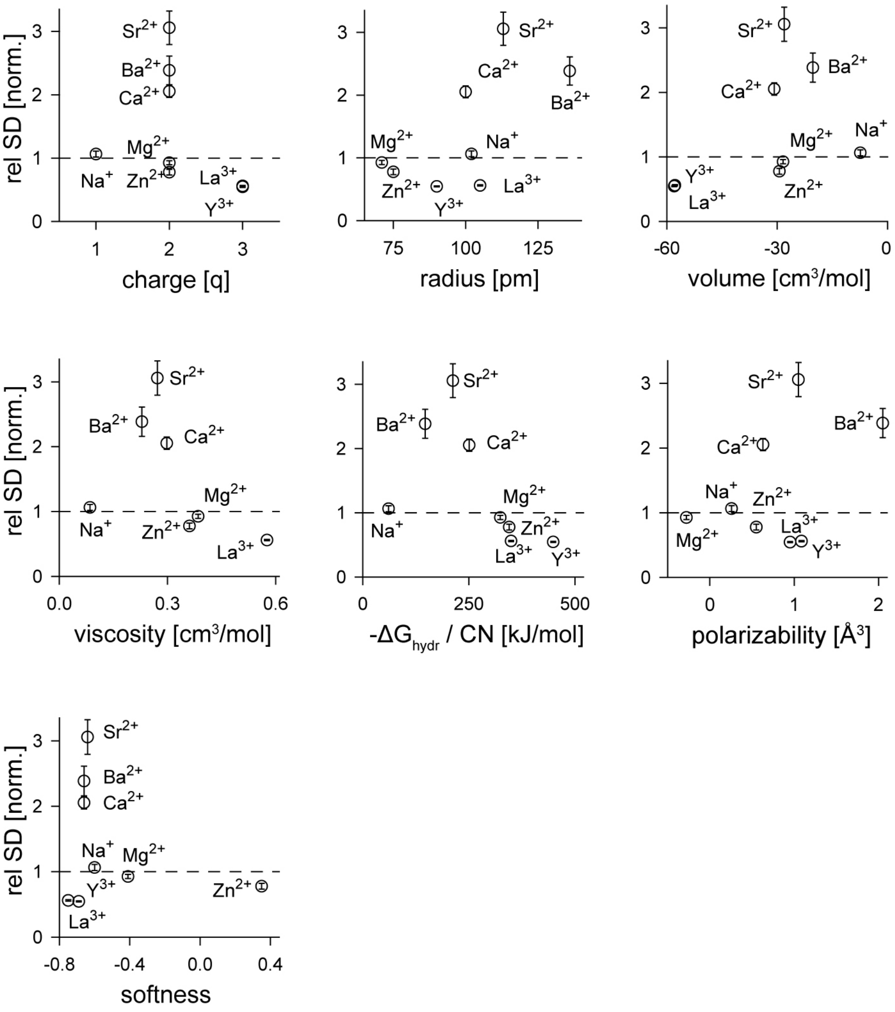

Supplementary Figure 5. Ion properties in relation to their effectiveness to cluster SNAP25 in native cell membrane sheets. The relative standard deviation (rel. SD) of the immunofluorescence intensity (normalized to the control condition, indicated by the dashed line; values are taken from Fig. 2b) is plotted in relation to the following ion properties: charge, crystal radius[4], ionic volume (according to the calculated standard partial molar volumes described in ref. [5]), the Jones–Dole viscosity B coefficient[4], Gibbs free energy of hydration[6] per coordinating water molecule (for references on the coordination numbers (CN) see ref. [7] (for Mg^{2+}, Ca^{2+} Sr^{2+}, Ba^{2+}), ref. [8] (for Na^{+} and Zn^{2+}), ref. [9] (for La^{3+}) and ref. [10] for (Y^{3+})), ion polarizability[4] and softness[4].

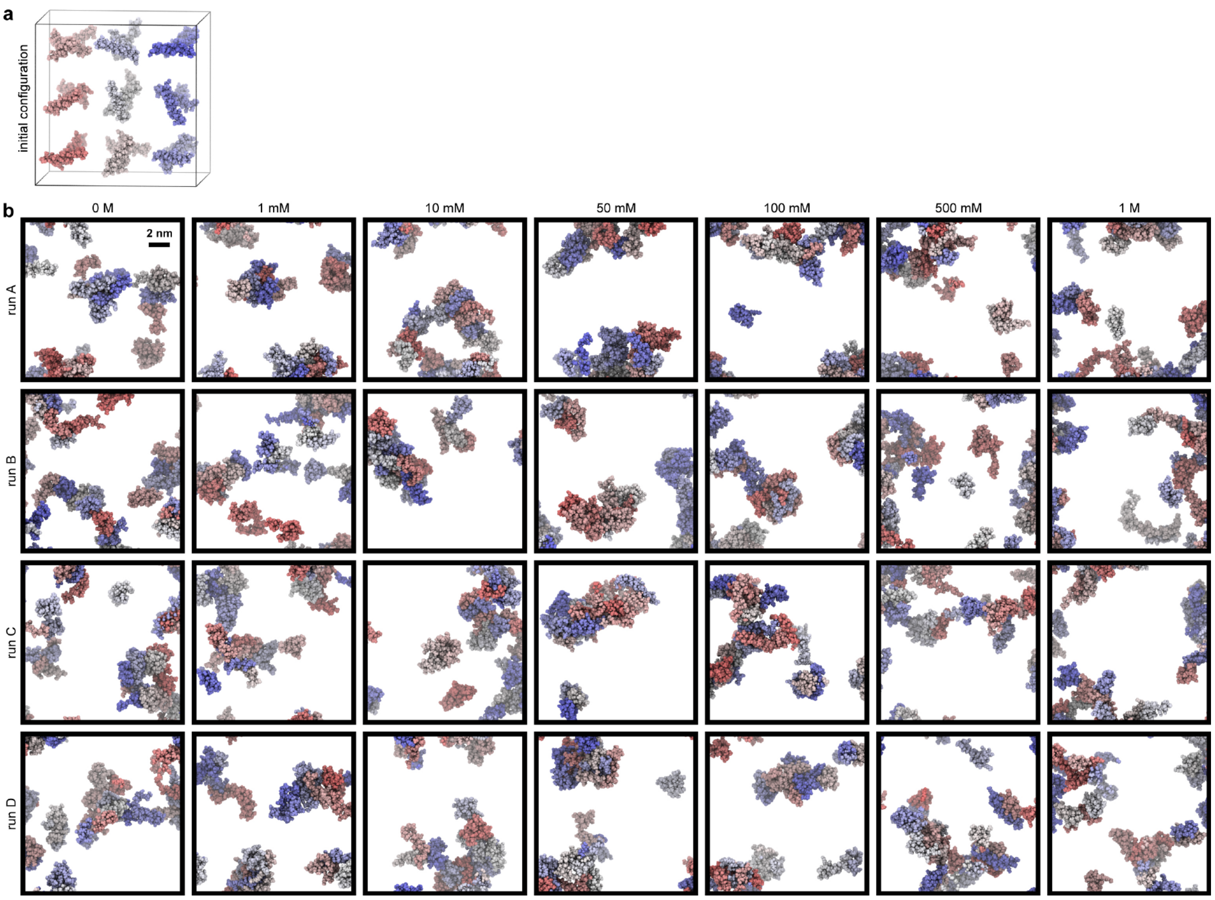

Supplementary Figure 6. Biphasic clustering behaviour in response to Ca^{2+} ions is observed for a SNAP25 peptide in molecular dynamics (MD) simulations.

(**a**) MD initial configuration of the 27 SNAP25 peptides solvated in an aqueous environment (160 mM NaCl; the example for 10 mM $CaCl_2$ after energy minimization is shown). Since water molecules are important ion interaction partners, they were explicitly modelled using TIP3P water[11] instead of treating the water as a continuous bulk with a defined electric permittivity. For clarity only the peptides (van der Waals representation) are shown while ions and water molecules are omitted. Peptides were coloured according to their position in the cubic simulation box using a red-grey-blue gradient. (**b**) MD system configurations after 100 ns unbiased simulation, illustrating the oligomeric states of the 27 SNAP25 peptides (coloured according to their starting position, see (**a**)). Due to periodic boundary conditions, molecules crossing simulation box borders are present at the respective opposite sides. In Fig. 3a, the final configuration of run A at 10 mM Ca^{2+} is shown from a different perspective for a better illustration of the formed oligomers.

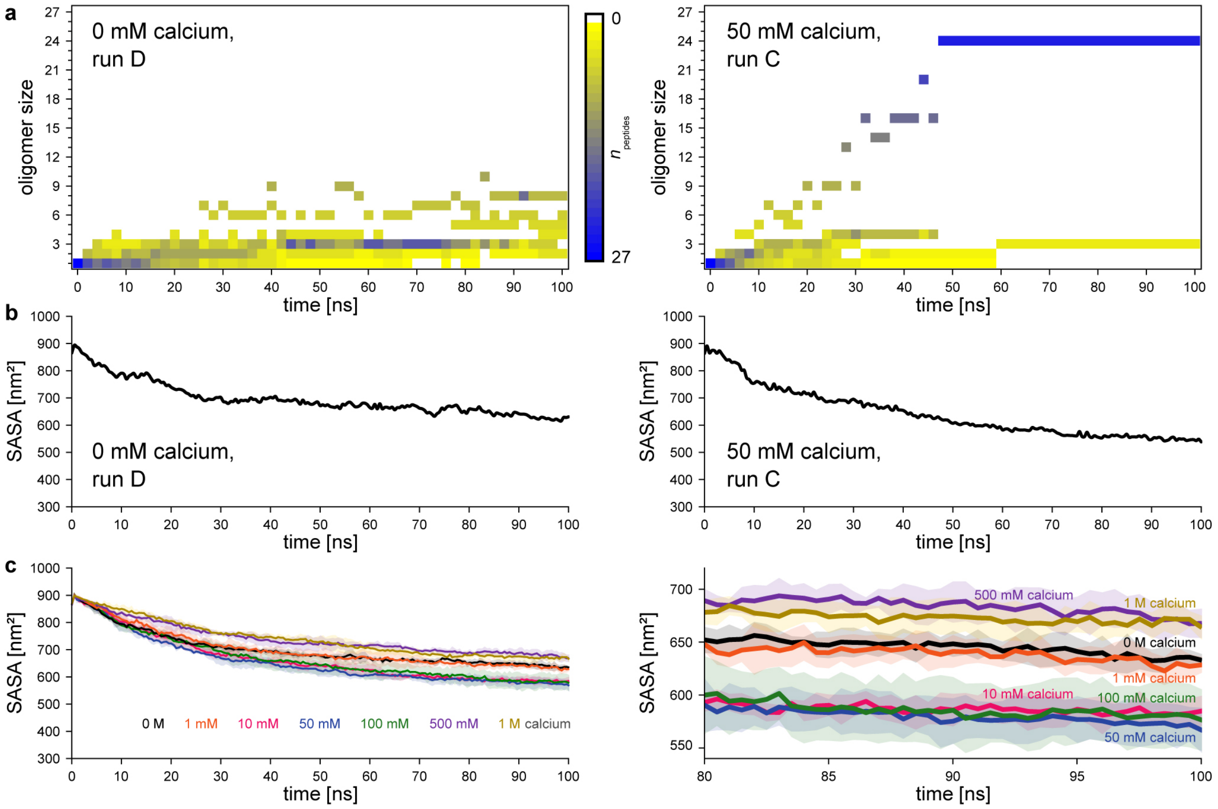

Supplementary Figure 7. Solvent accessible surface area (SASA) of oligomerizing SNAP25 peptides over time. The oligomeric state (**a**) and the SASA (**b**) of the peptides are shown for two individual runs for direct comparison of these two parameters. (**a**) Oligomer size distribution illustrated for one run at 0 mM calcium (left) and one run at 50 mM calcium (right) as function of time (generated using the GROMACS program gmx clustsize). Peptides within a distance of 0.35 nm were considered to be in physical contact. Colours indicate the number of peptides present in the respective oligomeric state (see also lookup table; note that at the beginning all 27 peptides are present as monomers). In the simulation run at 0 mM calcium (left), a mixture of middle sized oligomers is observed after 40 ns. In the run at 10 mM calcium (right) one 24-mer and one trimer form after 60 ns and remain stable until the end of the simulation time. (**b**) Corresponding SASA time courses of the runs shown in (**a**). Note that although the degree of oligomerization does not change in the 10 mM calcium run (right) after 60 ns, the SASA still decreases. This indicates that the SASA is sensitive to both initial contact establishment as well as further condensation/tighter packing of oligomers. (**c**) Mean SASA of the four simulation runs per calcium condition monitored over the entire simulation time range (left). The magnified view (right) shows the SASA during the last 20 ns, which were considered for further analysis. Coloured areas indicate the s.e.m.

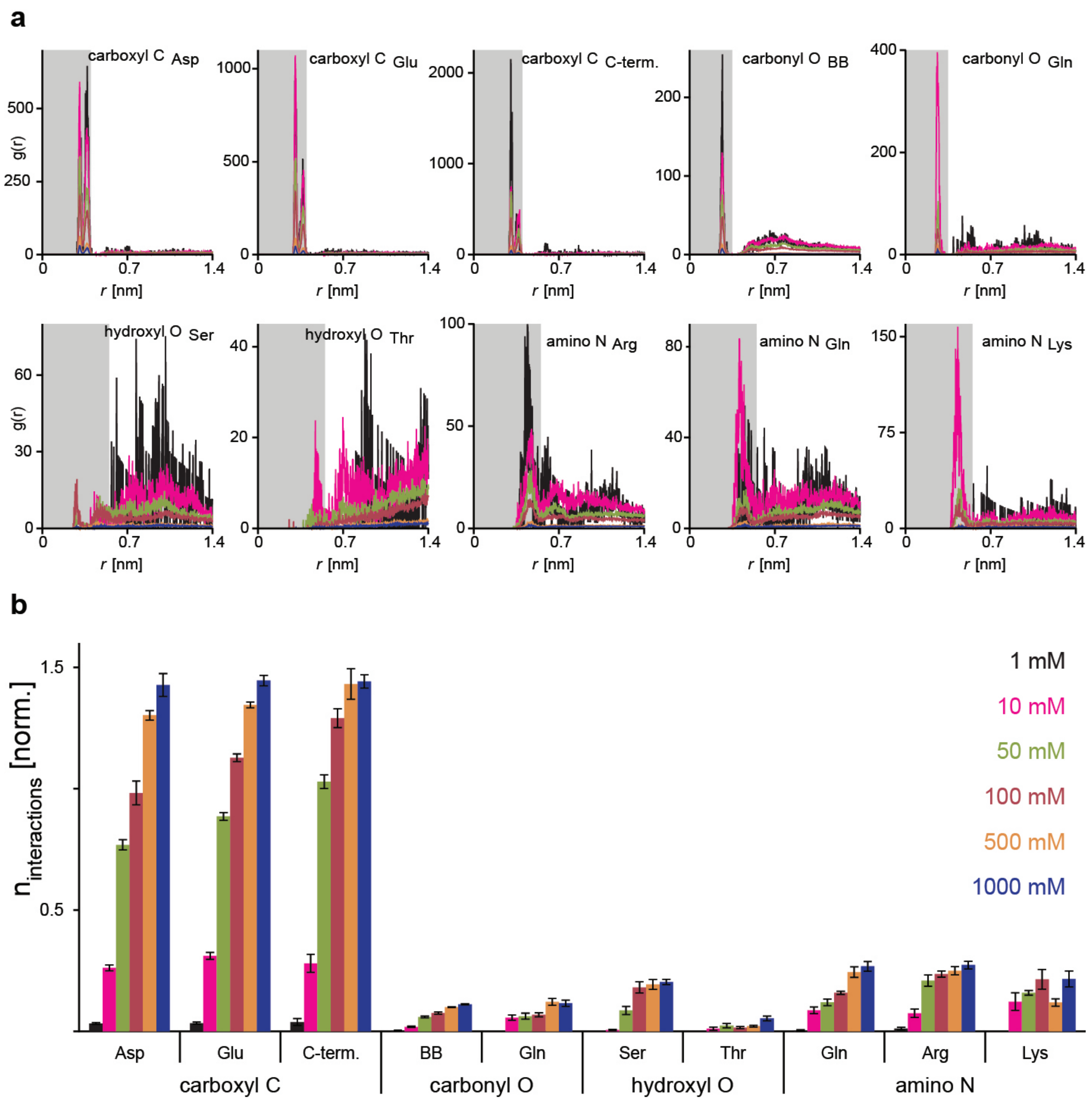

Supplementary Figure 8. Identification of preferred Ca^{2+} interaction partners. (**a**) Radial distribution functions (RDFs) g(r) for Ca^{2+} and respective atoms of putative interaction partners of the protein, i.e. charged and polar amino acid side chains, the protein C-terminus and the backbone carbonyl groups. For arginine, both primary amino nitrogen atoms of the guanidinium group were considered. Coloured curves indicate the different Ca^{2+} concentrations (see legend in (b)). Based on the RDF, distance thresholds, describing direct Ca^{2+} interactions with the respective protein atom, were defined for further analysis (grey area). (**b**) Average number of direct Ca^{2+} interactions with respective interaction partners per Ca^{2+} concentrations (n = 4 multi-copy runs, only the last 20 ns were considered), normalized to the number of interaction partners. Values are given as mean ± s.e.m.

**MD system**	**0 mM Ca^{2+}**	**1 mM Ca^{2+}**	**10 mM Ca^{2+}**	**50 mM Ca^{2+}**	**100 mM Ca^{2+}**	**500 mM Ca^{2+}**	**1000 mM Ca^{2+}**
$\mathbf{n_{atoms}}$	358806	358791	358659	358053	357303	351279	343743
$\mathbf{n_{peptides}}$	27	27	27	27	27	27	27
$\mathbf{n_{H2O}}$	115086	115078	115012	114709	114334	111322	107554
$\mathbf{n_{Ca2+}}$	0	3	25	126	251	1255	2511
$\mathbf{n_{Na+}}$	564	564	564	564	564	564	564
$\mathbf{n_{Cl-}}$	402	408	452	654	904	2912	5424

Supplementary Table S1. Molecular dynamics system data. The table shows the total number of atoms, peptides, water molecules, and ions present in the simulated MD systems.

Supplementary References

1. Zilly, F. E. *et al.* Ca2+ induces clustering of membrane proteins in the plasma membrane via electrostatic interactions. *EMBO J.* **30,** 1209–1220 (2011).
2. Knowles, M. K. *et al.* Single secretory granules of live cells recruit syntaxin-1 and synaptosomal associated protein 25 (SNAP-25) in large copy numbers. *Proc. Natl. Acad. Sci.* **107,** 20810–20815 (2010).
3. Halemani, N. D., Bethani, I., Rizzoli, S. O. & Lang, T. Structure and Dynamics of a Two-Helix SNARE Complex in Live Cells. *Traffic* **11,** 394–404 (2010).
4. Marcus, Y. *Ion properties*. (Marcel Dekker, 1997).
5. Marcus, Y. Thermodynamics of solvation of ions. Part 6.—The standard partial molar volumes of aqueous ions at 298.15 K. *J Chem Soc Faraday Trans* **89,** 713–718 (1993).
6. Marcus, Y. A simple empirical model describing the thermodynamics of hydration of ions of widely varying charges, sizes, and shapes. *Biophys. Chem.* **51,** 111–127 (1994).
7. Peschke, M., Blades, A. T. & Kebarle, P. Hydration Energies and Entropies for Mg^{2+}, Ca^{2+}, Sr^{2+}, and Ba^{2+} from Gas-Phase Ion−Water Molecule Equilibria Determinations. *J. Phys. Chem. A* **102,** 9978–9985 (1998).
8. Persson, I. Hydrated metal ions in aqueous solution: How regular are their structures? *Pure Appl. Chem.* **82,** (2010).
9. Habenschuss, A. & Spedding, F. H. The coordination (hydration) of rare earth ions in aqueous chloride solutions from x-ray diffraction. III. SmCl3, EuCl3, and series behavior. *J. Chem. Phys.* **73,** 442 (1980).
10. Buz'ko, V. Y., Sukhno, I. V., Buz'ko, M. B., Polushin, A. A. & Panyushkin, V. T. Study of the structure and stability of aqua complexes Y(H2O) n 3+ (n = 1–10) by Ab initio methods. *Russ. J. Inorg. Chem.* **51,** 1276–1282 (2006).
11. Mark, P. & Nilsson, L. Structure and Dynamics of the TIP3P, SPC, and SPC/E Water Models at 298 K. *J. Phys. Chem. A* **105,** 9954–9960 (2001).
